# Nicotine exposure potentiates lung tumorigenesis by perturbing cellular surveillance

**DOI:** 10.1038/s41416-020-0730-0

**Published:** 2020-01-31

**Authors:** Qiang Zhang, Suthakar Ganapathy, Hava Avraham, Takashi Nishioka, Changyan Chen

**Affiliations:** 1 The First Affiliated Hospital of Nanchang University, Nanchang, P.R. China; 2 Center for Drug Development, Northeastern University, Boston, MA USA; 3 Tohoku University Graduate School of Dentistry, Sendai, Japan

**Keywords:** Health sciences, Molecular medicine

## Abstract

**Background:**

Nicotine is a major tobacco component and found at circulating concentrations in smokers’ bloodstreams. Although considered a non-carcinogenic substance, nicotine rapidly defuses to tissues after being inhaled, inviting effects on cellular physiology, particularly in the lung. Widespread increased use of nicotine-based e-cigarettes, especially in younger adults, creates an urgent need for improved understanding of nicotine’s potential to impact human health.

**Methods:**

Biological and biochemistry methods were used to interrogate the potential for nicotine to weaken the genetic integrity of murine and human-lung epithelial cells.

**Results:**

We demonstrate that nicotine potentiates the growth of the lung epithelial cells in a dose–response fashion. Nicotine elicits an acute increase in reactive oxygen species (ROS), which persists at moderately high levels throughout the duration of nicotine exposure. The aberrant increases in ROS appear to induce ER stress and UPR activation, as reflected by BIP upregulation and PERK phosphorylation. Furthermore, prolonged nicotine exposure interferes with p53 function triggered by sodium arsenite. Unless p53 is suppressed, persistent nicotine exposure does not induce colony formation by lung epithelial cells in soft agar.

**Conclusion:**

The data suggest that nicotine treatment, by perturbing intracellular redox state and altering p53 function, can create a pro-tumorigenic environment in lung epithelium. The results suggest caution in using nicotine replacement therapies and e-cigarettes.

## Background

A well-recognised environmental biohazard, tobacco smoke, when inhaled, is associated with various human diseases, especially lung illnesses and malignancies.^1–3^ Nicotine, one of the major components in tobacco smoke, carries significant abuse/addiction potential, though not itself carcinogenic, and rapidly diffuses into various tissues of human bodies via the circulation.^4^ Although emerging evidence suggests a pathogenic link between nicotine and increased susceptibility of cells to malignancies, the underlying mechanisms remain ill-defined. Nicotine, by binding to nicotine acetylcholine receptors (nAchRs), can activate several intracellular mitogenic signalling pathways that promote cell growth, angiogenesis and other tissue responses.^4–7^ For example, src and Raf in non-small-cell lung (and other cancer) cells were phosphorylated upon nicotine treatment, resulting in E2F activation and perturbation of cell cycle restrictions. Subsequently, genes regulating cell growth or invasion were upregulated.^8–12^ Nicotine can also promote pro-inflammatory signalling in the vasculature that accelerates atherosclerotic plaque formation.^13,14^

Expressed in most non-neuronal or somatic cells including lung cells, nACHRs, when activated by nicotine binding, can elicit varied changes in cellular physiology by inciting/modulating downstream intracellular signalling pathways.^15–17^ Nicotine augments the levels of choline acetyltransferase and vesicular acetylcholine (Ach) transporter in human bronchioalveolar carcinoma cells to increase Ach production and promote growth-related activities.^18,19^ Nicotine can also alter ACh signalling in lung cells, which influences muscarinic control of mitogen-regulated activities.^18,19^ Furthermore, nAChR-mediated signalling is a critical promotor of breast cancer development, such that nAChR blockade abolished nicotine-mediated growth promotion in vitro and in vivo.^9^ Nicotine is capable of mobilising src, Raf/MEK/ERK and PI3K/Akt pathways to activate the transcription of genes involved in promoting cell growth in the lung.^8,9,20^ By affecting mitochondrial electron transport, nicotine can also mitigate chemotherapy-induced apoptosis.^21–24^ In aggregate, these data suggest that nicotine exposure can attenuate growth restriction in non-neuronal cells.

Collectively, ROS include the inorganic hydrogen peroxide (H_2_O_2_), and the hydroxyl and superoxide anion-free radicals. Physiologically, ROS are signalling molecules involved in normal cellular functions.^25,26^ Several enzymatic and non-enzymatic mechanisms exist for keeping ROS levels within physiological limits, and preventing cell- and tissue-damaging oxidative stress, an imbalance favouring ROS production in excess of the means to inactivate them. NADPH oxidases plays a crucial role in the upregulation of ROS in cancer cells, since stimulation of this enzyme family generates a burst of superoxide anion radicals as the initial product that can be catalytically dismutated to H_2_O_2_ by catalase.^27^ In pancreatic cancer cells, nicotine treatment was shown to affect ROS signalling to dysregulate cell growth.^28^

Persistent increases in ROS are often detected in cancer cells, which causes cellular or endoplasmic reticulum (ER) stress.^29,30^ Chaperones in the ER help proteins to fold properly and translocate to other cellular compartments while preventing aberrant formation/accumulation of defective species within the organelle.^31,32^ BIP is one such ER chaperone, its synthesis stimulated by cellular stresses.^29,30^ Accordingly, increased BIP expression is an indicator of ER stress. Although the ER compartment can accommodate acutely appreciable levels of partially folded proteins, persistent accumulation of aberrant folded proteins activates UPR and upregulates PERK and other “stress sensors”.^33^ In the lung, the impact of ER stress and UPR activation elicited by persistent nicotine exposure on lung-cell (patho)-physiology remains to be defined.

In this study, we demonstrate that long-term nicotine exposure upregulated ROS in murine and human-lung epithelial cells, and triggers ER stress/UPR activation coincident with attenuated p53 function. Persistent nicotine exposure was transformational for lung epithelial cells when p53 was lost. Our data reveal the tumorigenic potential of nicotine and attribute it to a cancer risk once tumour surveillance systems are compromised.

## Methods

### Cells and reagents

Human-lung epithelial BEAS-2B and murine lung epithelial LA4 cells were purchased from ATCC (Manassas, VA). The cells were grown in Dulbecco’s Modified Eagle’s minimal essential medium (DMEM) supplemented with 10% heat-inactivated foetal bovine serum (Invitrogen, CA), 100 units/ml penicillin and 100 μg/ml streptomycin (Invitrogen) under a 5% CO_2_-humidified atmosphere at 37 °C. Nicotine (Sigma, MO) was administered from stock solutions in DMEM. Antibodies against p-p53, phor-ser-15-p53, phor-PERK, caspase-3, PERK, α-tubulin and β-actin were purchased from Cell Signaling Technology (Danvers, MA). BIP antibody was from Santa Cruz Biotechnology (Santa Cruz, CA).

### Cell growth analyses

After synchronisation by serum starvation for 24 h, cells were cultured in DMEM containing different concentrations of nicotine. Subsequently, cells were harvested daily and stained with trypan blue (Sigma, MO). The number of viable cells was determined by counting with a haemocytometer. For long-term growth, cells (1000 cells/dish) were grown under the same conditions, and the medium was changed every third day. Fourteen days later, the cultures were fixed and stained with crystal violet (Sigma, MO). Colonies were counted under a microscope.

### ROS analysis

Treated or untreated cells were washed with ice-cold PBS and resuspended in 5 μg/ml of 2′,7′-dichlorodihydrofluorescein diacetate (DCF) (Thermo Fisher Scientific, MA) at 37 °C for 30 min. The samples were incubated for 10 min at room temperature and analysed immediately by using a flow cytometer.

### Immunoblotting

Cells were harvested, washed with 1×PBS twice and lysed in lysis buffer (50 mM Tris-HCl, pH 8.0, 150 mM NaCl, 1% Triton-X114, 0.5% sodium deoxycholate and 0.1% sodium dodecyl sulfate, supplemented with protease and phosphatase inhibitor cocktails) (Thermo Scientific). Protein concentration was determined with a Bradford dye-binding assay (Sigma, MO). Subsequently, proteins in the cell lysates were resolved on 10% SDS-PAGE gels (Invitrogen) and transferred to Immobilon-P membranes (Millipore). After blocking with 5% non-fat milk in TBS-Tween, the membranes were probed with specified antibodies. Blots were stripped and re-probed for loading controls. The intensities of proteins being tested were determined densitometrically with the Image J program.

### Luciferase assay

Cells were transiently transfected with an empty *luc* or *luc-p53* construct for 48 h prior to treatments. Subsequently, cell lysates were prepared, and luciferase activity was quantified by using an assay kit (Promega Corp., WI) according to the manufacturer’s protocol.

### DNA fragmentation analysis

Flow cytometry was performed using a FACScan (BD Biosciences). Data analysis was performed using the Cell-Fit software program (BD Biosciences). Cell-Fit receives data from the flow cytometer and provides real-time statistical analysis, computed at 1-s intervals, and also discriminates doublets or adjacent particles. In brief, after treatment, the cells were harvested and then fixed in 70% cold ethanol. Afterwards, cells were stained with 0.01 μg/ml of propidium iodide containing 1.5 ng/ml of RNase. Cells with sub-G_0_–G_1_ DNA contents after staining with propidium iodide were counted as apoptotic cells. Cellular DNA content was quantified using the FACScan.

### Soft agar assay

Cells or *E6* transfectants (5000/dish) were seeded in 0.5% low-melting-point agarose in DMEM growth medium in the presence or absence of nicotine or sodium arsenite, and layered onto 0.8% agarose in DMEM growth medium. Cell dishes were kept in the culture incubator for 100 days. Through this incubation period, 0.3 ml of fresh medium with or without nicotine or sodium arsenite was added over the soft agar every 3 days. Formed colonies were then counted under a light microscope.

### Statistical analysis

Averages and standard deviations of the results of the experiments were computed. Standard deviations are denoted as error bars in the figures. *p* value of <0.05 in the Student’s *t* test was considered significant.

## Results

### Nicotine treatment promotes the growth of human or murine lung epithelial cells

**Fig. 1:**
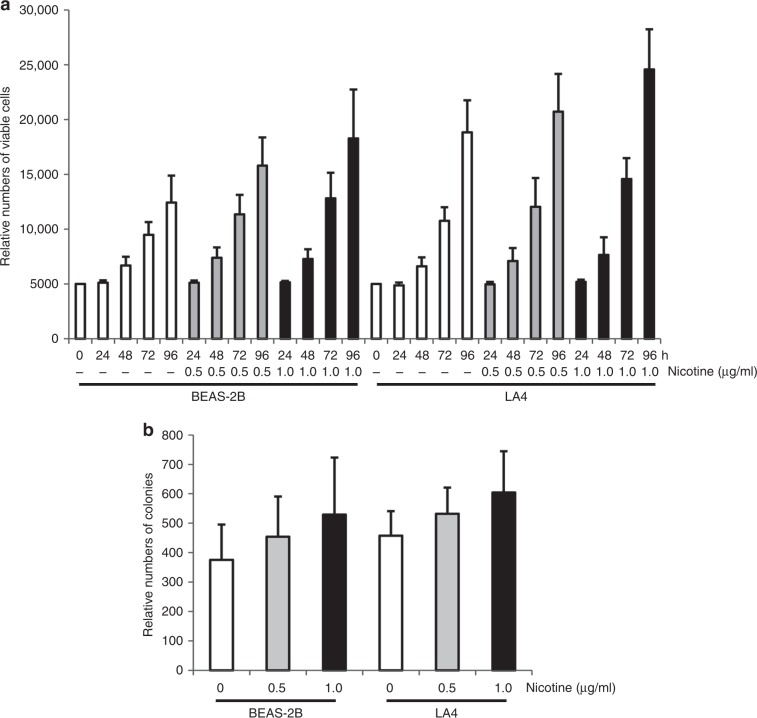
Nicotine promotes growth of lung epithelial cells. **a** After being synchronised by serum starvation, human BEAS-2B or murine LA4 lung epithelial cells were cultured in medium containing different concentrations of nicotine for 4 consecutive days. Subsequently, cells were counted daily. Data are means + SD from five independent experiments (*n* = 5, *ρ* < 0.05). **b** Cells were cultured under the conditions as described above for 14 days. Subsequently, the colonies were counted. Data are means + SD from five independent experiments (*n* = 5, *p* < 0.05).

Although nicotine is an addictive substance and not a conventional carcinogen, this major tobacco constituent is motigenic.^34^ To investigate the role of nicotine in cell growth, we employed human-lung epithelial BEAS-2B and murine LA4 cell lines as study objects. After synchronisation by serum starvation, the cells were exposed under culture conditions to varying concentrations of nicotine,^8,23^ for 4 consecutive days (Fig. 1a). Untreated cells represented the normal growth pattern. The growth rates of both cell lines treated with nicotine were slightly potentiated in a concentration–response manner. The long-term effect of nicotine exposure on cell growth was also examined by colony formation assay after 14 days. Consistently, nicotine concentration dependently increased colony formation by BEAS-2B and LA4 cells (Fig. 1b).

### ROS state is potentiated, and ER stress/UPR are induced in nicotine-treated lung epithelial cells

**Fig. 2:**
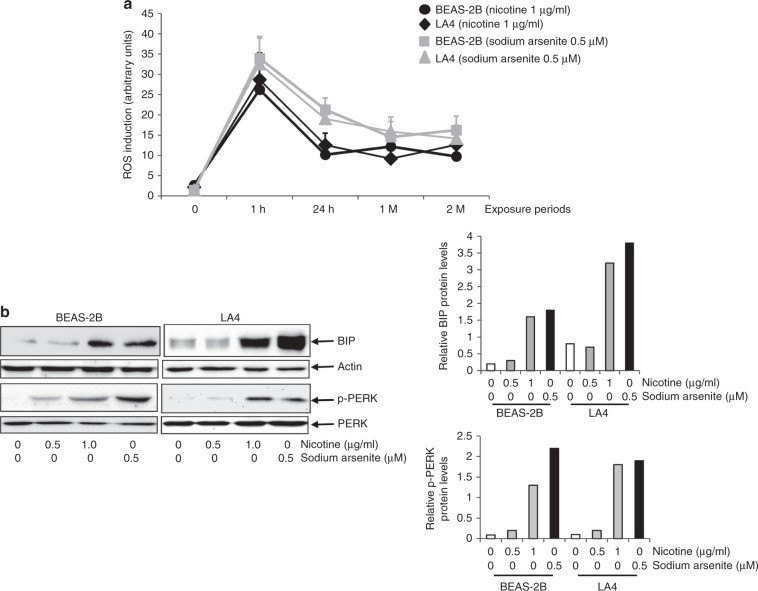
Prolonged nicotine exposure perturbs ROS state and induces ER stress/UPR in lung epithelial cells. **a** Cells were treated with 1.0 μg/ml nicotine or 0.5 μg/ml sodium arsenite for different time periods. Cellular ROS levels were measured. Data are means + SD from five independent experiments (*n* = 5, *p* < 0.01). **b** BIP expression and PERK phosphorylation after cell exposure to nicotine or sodium arsenite for 1 month, as analysed by immunoblotting (left panel). Relative BIP and p-PERK expression in response to designated treatments are plotted (right panels). *β*-actin and PERK are loading controls.

By damaging cellular macromolecules (DNA, RNA, lipids and proteins), oxidative stress that is caused by the aberrant increase in ROS beyond the capacity of intrinsic detoxifying systems to remove/inactivate them, has been linked causatively to cell transformation and tumorigenesis.^25,26,35,36^ Because nicotine can be involved in free radical-mediated signalling,^28^ ROS levels in BEAS-2B and LA4 cells in response to nicotine treatment were quantified (Fig. 2a). Sodium arsenite at a low 0.5 μM concentration induces tumorigenesis under conditions in which ROS increases and ER stress were observed, such that this metal toxin could be used as a positive control.^37^

Nicotine (1.0 μg/ml) dramatically increased ROS production by murine and human-lung epithelial-cell lines within 1 h post treatment. The nicotine-induced potentiation of cellular ROS formation declined moderately by 24 h of nicotine exposure and remained at a steady level thereafter. Although the magnitude of ROS production in cells treated with 0.5 μM sodium arsenite was greater than that induced by 1.0 μg/ml nicotine, the kinetics of ROS production in response to these two agents were similar. Thus, the data indicate that the tobacco constituent nicotine can upregulate and sustain a heightened ROS production in lung epithelial cells.

Persistent increases in cellular ROS often trigger ER stress and UPR activation, which plays an important role in tumorigenesis.^29–32^ During ER stress and subsequent UPR activation, BIP (an ER chaperone protein) may be upregulated, and UPR sensors (like PERK) are phosphorylated.^29–32^ To determine whether lung epithelium responds in this manner to nicotine-induced oxidative stress, BIP expression was analysed in BEAS-2B and LA4 cells treated with either sodium arsenite (positive control) or nicotine at different doses for 1 month (Fig. 2b, left, upper two panels). We found that nicotine increased BIP expression in BEAS-2B and LA4 lung epithelial-cell lines to a similar extent as did sodium arsenite. Phosphorylation of PERK was also analysed in response to arsenite or nicotine treatment (Fig. 2b, left, lower two panels). Phosphorylated PERK (p-PERK) was detected consistently in lung epithelial cells, and cell exposure to either agent induced several-fold increases in p-PERK expression. These results suggest that nicotine exposure can elicit ER stress and activate UPR in lung epithelium, which could help establish a permissive microenvironment for tumour initiation and development.

### Nicotine exposure interferes with p53 function in human or murine lung epithelial cells

**Fig. 3:**
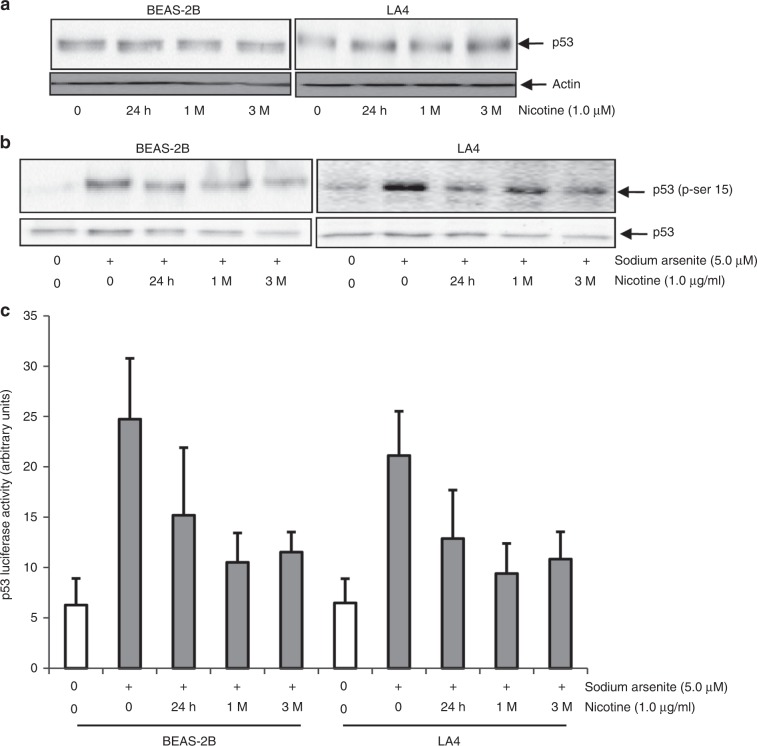
Nicotine interferes with p53 activity in lung epithelial cells. **a** After treatment with 1.0 μg/ml nicotine for various durations, cell lysates were prepared and immunoblotted with anti-p53 antibody. Actin was the loading control. **b** Cells were exposed to 1.0 μg/ml nicotine for various durations, and then treated with sodium arsenite (5.0 μM). Subsequently, cell lysates were prepared and subjected to immunoblotting with the anti-phor (ser-15)-p53 antibody. p53 is the loading control. **c** Cells were exposed to nicotine for various durations. Luciferase reporter construct inserted with *p21* promoter that contains p53-binding sequence was then transfected into the cells. Forty-eight hours later, the cells were treated with sodium arsenite, and then subjected to the luciferase assay. Data are means ± SD from three independent experiments (*n* = 3; *p* < 0.05).

Tumour suppressor p53 is a crucial safeguard for cell cycle progression, and protects against ROS-induced genome damage.^35,36^ To examine the potential of nicotine to alter p53 in lung epithelium, BEAS-2B and LA4 cells were treated with nicotine for various time periods, after which p53 expression was analysed by immunoblotting (Fig. 3a). Similar baseline expressions were detected in untreated or treated cells, indicating that nicotine treatment did not affect p53 expression. To determine if nicotine affected p53 activation, the lung epithelial-cell lines were first exposed to nicotine for different time periods, and then treated with a high-dose sodium arsenite (5.0 μM) that had been shown to induce p53 by triggering a genotoxic response.^35,36^ Subsequently, phosphorylation of p53 (canonically, at its serine-15 residue) was analysed by immunoblotting (Fig. 3b). In both cell lines, sodium arsenite alone elicited marked p53 phosphorylation as a cytoprotective, tumour-suppressive stress response. Nicotine attenuated the sodium arsenite-mediated p53 phosphorylation. To define further the effect of nicotine on p53, cells either exposed to nicotine or not for different periods were transiently transfected with a luciferase construct inserted with *p21* promoter sequence that contains the p53-binding site. Forty-eight hours later, the cells were treated with sodium arsenite (Fig. 3c). The p53-regulated promoter activity was dramatically increased by sodium arsenite treatment alone, and this increase was attenuated by nicotine. These data are in good agreement that the tumour-suppressive effect of p53 function in lung epithelial cells can be reduced by nicotine.

### Nicotine exposure desensitises the cells to sodium arsenite-induced apoptosis

**Fig. 4:**
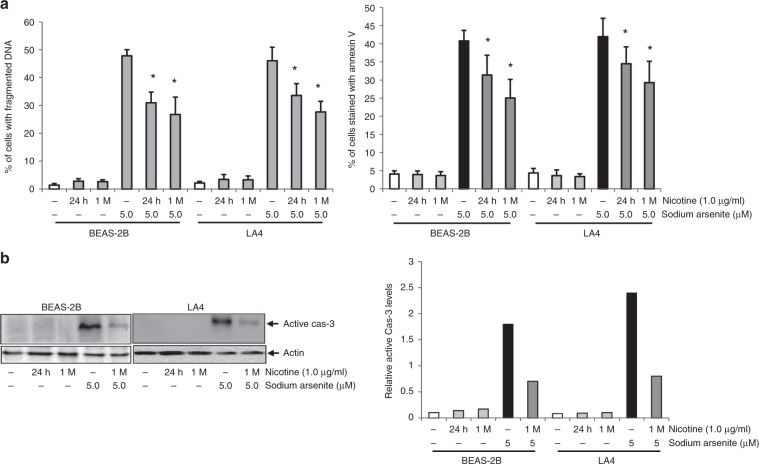
Nicotine desensitises sodium arsenite-induced apoptosis in lung epithelial cells. **a** After exposure to nicotine for different durations, cells were treated with sodium arsenite for 24 h. Subsequently, DNA fragmentation was assessed (left panel). After the same treatments, an Annexin V apoptotic assay was performed (right panel). Data are means ± SD over five independent experiments (*n* = 5; *p* < 0.01). **b** Following the same treatments described, cell lysates were prepared and subjected to immunoblotting to assay active cas-3 expression (left panel), quantification of which is given in the right panel as relative fold increase. *β*-actin is the loading control.

The influence of nicotine on sodium arsenite-induced apoptosis was examined. Lung epithelial cells either exposed to nicotine or not for different time periods were treated with sodium arsenite (5.0 μM), and a DNA fragmentation assay was then performed (Fig. 4a, left panel). Approximately 40% of the cells underwent apoptosis after a 24-h sodium arsenite treatment. Nicotine exposure alone for 24 h or 1 month did not affect cell viability. However, sodium arsenite-induced apoptosis in both BEAS-2B and LA4 lung-cell lines treated with nicotine was attenuated in a time-dependent fashion. A similar result was obtained by using the Annexin V-FITC apoptotic assay (Fig. 4a, right panel).

Caspase-3 (cas-3) is a major player in promoting apoptosis, during which this protease is cleaved to a small and active fragment of about 15–19 kD.^38^ To characterise in more detail the effect of nicotine on apoptosis in lung epithelium, BEAS-2B and LA4 lung-cell lines, either exposed to nicotine for various times or not, were treated with sodium arsenite, and expression of active cas-3 was then analysed by immunoblotting and quantified (Fig. 4b). As positive control, the active form of cas-3 in cells treated with sodium arsenite was unambiguously revealed by the antibody. No active form of cap-3 was detected in the cells treated with nicotine alone. However, nicotine significantly attenuated sodium arsenite-induced, active cas-3 expression in both human BEAS-2B and murine LA4 cells, further evidence that nicotine can antagonise sodium arsenite-induced apoptosis in lung epithelium.

### Persistent exposure to nicotine potentiates lung epithelial-cell transformation

**Fig. 5:**
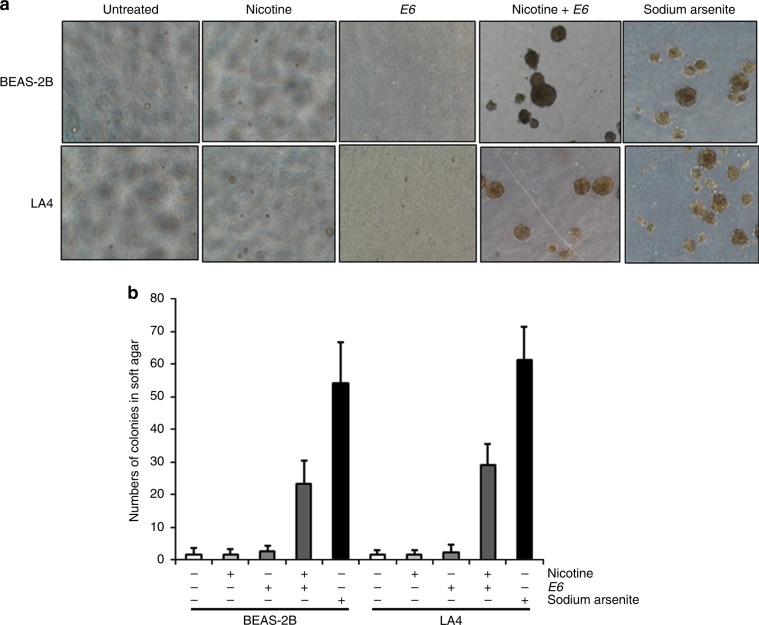
Long-term nicotine treatment potentiates transformation of lung epithelial cells. Cells or *E6* transfectants were grown in soft agar in the presence or absence of nicotine (1.0 ug/ml) or sodium arsenite (0.5 uM). One-hundred days later, representative photographs of colony examples were taken (**a**). The numbers of colonies in each dish were counted and plotted (**b**). Data are means ± SD from three independent experiments (*n* = 3; *p* < 0.05).

The potential of nicotine to induce transformation of lung epithelial cells was tested, by using a soft agar assay. The cells were stably transfected with *E6* that inhibits p53. Subsequently, the cells and *E6* transfectants were cultured in soft agar for 100 days with or without either 1.0 μg/ml nicotine or sodium arsenite (0.5 μM), the latter treatment known to induce cells to undergo transformation.^37^ Afterwards, the colonies grown in soft agar were imaged (Fig. 5a) and counted (Fig. 5b). At 100 days, a few BEAS-2B or LA4 cells formed colonies when grown in soft agar whether exposed to nicotine or not. Inhibition of p53 alone did not promote colony formation. Sodium arsenite treatment permitted the cells to form colonies in soft agar, as expected. Notably, some of *E6* transfectants also formed colonies in soft agar containing nicotine. These data suggest that nicotine synergises with p53 loss to promote transformation of the lung epithelial cells.

## Discussion

Not a conventional carcinogen, nicotine, tobacco alkaloid inhaled and absorbed during cigarette smoking, carries substance-abuse potential that can lead to addiction. Due to its non-carcinogenic nature and pharmacological activity as a nicotinic cholinergic agonist in the central and peripheral nervous system, nicotine has been used therapeutically as, for example, a smoking-cessation aid and for relieving chronic pain.^1–4^ However, the extent to which nicotine might adversely affect human health remains unclear, as do any underlying pathogenic mechanisms at the cellular level. It has been reported that nicotine affected cell cycle restriction in murine lung epithelial cells by deregulating cyclin D1 expression, which perturbed cell growth.^5^ In this investigation, we focused on examining the effects of nicotine on redox state and its potential to alter the physiology of human and murine lung epithelial cells in a manner that could support tumorigenesis. First, we showed that nicotine treatment promoted the growth of the epithelial cells in a concentration–response fashion. Nicotine elicited an initial, transient ROS increase in the cells, which was attenuated, yet persisted at a moderately high level, during prolonged nicotine treatment when ER stress and UPR activation occurred. Nicotine also interfered with sodium arsenite-mediated p53 activation. However, prolonged nicotine treatment alone was incapable of initiating transformation of lung epithelial cells unless p53 was inhibited. Overall, the concomitant conditions of disrupted pro-oxidative redox state (“oxidative stress”) and attenuated p53 function induced by nicotine provide a microenvironment favouring tumorigenesis in lung epithelium.

Concentrations of nicotine observed in the bloodstreams of heavy smokers were used in this study.^8,23^ Our data revealed that nicotine, regardless of transient or persistent exposure, was incapable of inducing p53 activation in the murine- and human-lung epithelial-cell lines. However, p53 function was limited by nicotine treatment, as indexed by nicotine’s attenuation of sodium arsenite-induced p53 activation. We previously demonstrated that the genetic stability of rat lung epithelial cells following long-term nicotine treatment was perturbed.^20^ In response to nicotine exposure, the *DHFR* gene was amplified, and MTX-resistant colonies were formed, which seems responsible for nicotine-induced deregulation of genetic stability. In conjunction with our previous study, our current findings added significantly more evidence and mechanistic detail to support the proposition that persistent nicotine intake, perhaps by mitigating tumour-suppressive mechanisms, weakens the genetic integrity of cells, and thereby represents a human-health hazard.

At physiological levels, ROS act as intracellular signal transducers that regulate diverse cellular activities.^25,26^ In response to abnormal mitogenic or tumorigenic stimulation, supraphysiological increases in ROS tone can establish the condition of oxidative stress, which may help induce cellular transformation or oncogenesis by damaging cytoskeletal structure to allow cellular malignant progression or invasion. Epidermal growth factor stimulation of rat PC12 cells elicited an increase in ROS production, and upregulation of mitogenic signalling pathways.^39^ Oncogenes (such as *myc* or *ras)*, by dysregulating intracellular redox tone and inciting oxidative stress, were able to damage DNA structure, resulting in disrupted genetic stability, and predisposing cells to malignant changes.^40,41^ ROS are also principal effectors for arsenic-induced, uncontrollable cell proliferation involving p53 as a molecular target.^35,36^ The results presented here demonstrate that nicotine exposure interferes with sodium arsenite-induced p53 activation in lung epithelial cells. Further study is required to determine the precise relationship between interference of p53 function and disruption of redox balance by nicotine in lung epithelium.

Aberrant or persistent accumulation of unfolded or misfolded proteins in the ER caused by adverse changes in protein or lipid metabolism can elicit chronic UPR activation, a component of ER stress that often triggers cytoprotective or cytotoxic cell responses. It is well known that oxidative stress plays an obligatory role in inducing ER stress and UPR activation.^29,30^ For example, in response to TNF stimulation, NF-κB and JNK functioned synergistically to potentiate cellular ROS production, and lead to damaging the homeogenesis of cells.^42^ Oncogenes, by inducing oncogenic stress, can trigger ER stress and UPR activation during cancer genesis and development. For instance, inhibition of PKC triggered a significant ROS increase that disrupted the redox state in cancer cells harbouring oncogenic *K-ras*.^34^ Previously, we reported that long-term nicotine exposure activated src/Ras signalling in rat lung epithelial cells.^20^ In this study, we showed that BIP and PERK were activated by nicotine, responses that might lead to pro-tumorigenic changes in the cellular microenvironment. Thus, it is conceivable that upregulation of specific intracellular signalling pathways by nicotine changes epigenetic protective mechanisms (such as p53) in cells, exceeding the tolerance of the ER to withstand stress, but not enough to induce a full transformational process or rank tumorigenesis. Taken together, our data support the notion that nicotine, by potentiating cellular ROS production and impairing p53 function, changes the genetic landscape of cells. The possibility that tumour suppressors/promoters other than p53 might be involved in cellular changes underlying transformation remains to be investigated.

In summary, our investigation elucidates a molecular mechanism by which nicotine exposure perturbs the intracellular redox balance in murine and human-lung epithelial cells, which provides a microenvironment that favours cellular transformation or tumorigenesis. Importantly, when the genetic surveillance system is compromised by impaired p53, a nicotine tumorigenic property is unveiled, potentially sufficient for initiating a carcinogenic programme. Because nicotine replacement therapy is widely used as a therapeutic smoking-cessation aid,^43^ there is an urgent need for improving our understanding of adverse nicotine-mediated actions to help develop new strategies to protect human health.

## Data Availability

The data sets of the study are available from the corresponding author upon reasonable request.
